# Regulation of the Tumor-Suppressor Function of the Class III Phosphatidylinositol 3-Kinase Complex by Ubiquitin and SUMO

**DOI:** 10.3390/cancers7010001

**Published:** 2014-12-23

**Authors:** Christina Reidick, Fouzi El Magraoui, Helmut E. Meyer, Harald Stenmark, Harald W. Platta

**Affiliations:** 1 Biochemie Intrazellulärer Transportprozesse, Ruhr-Universität Bochum, Bochum 44801, Germany; E-Mails: christina.reidick@rub.de (C.R.); harald.platta@rub.de (H.W.P.); 2 Biomedical Research, Human Brain Proteomics II, Leibniz-Institut für Analytische Wissenschaften-ISAS, Dortmund 44139, Germany; E-Mails: fouzi.elmagraoui@isas.de (F.E.); helmut.e.meyer@isas.de (H.E.M.); 3 Department of Biochemistry, Institute for Cancer Research, Oslo University Hospital, Montebello, Oslo 0310, Norway; E-Mail: stenmark@ulrik.uio.no

**Keywords:** VPS34, Beclin 1, Ambra 1, ubiquitin, SUMO, autophagy, tumor suppressor

## Abstract

The occurrence of cancer is often associated with a dysfunction in one of the three central membrane-involution processes—autophagy, endocytosis or cytokinesis. Interestingly, all three pathways are controlled by the same central signaling module: the class III phosphatidylinositol 3-kinase (PI3K-III) complex and its catalytic product, the phosphorylated lipid phosphatidylinositol 3-phosphate (PtdIns3P). The activity of the catalytic subunit of the PI3K-III complex, the lipid-kinase VPS34, requires the presence of the membrane-targeting factor VPS15 as well as the adaptor protein Beclin 1. Furthermore, a growing list of regulatory proteins associates with VPS34 via Beclin 1. These accessory factors define distinct subunit compositions and thereby guide the PI3K-III complex to its different cellular and physiological roles. Here we discuss the regulation of the PI3K-III complex components by ubiquitination and SUMOylation. Especially Beclin 1 has emerged as a highly regulated protein, which can be modified with Lys11-, Lys48- or Lys63-linked polyubiquitin chains catalyzed by distinct E3 ligases from the RING-, HECT-, RBR- or Cullin-type. We also point out other cross-links of these ligases with autophagy in order to discuss how these data might be merged into a general concept.

## 1. The Concept of Phosphatidylinositol 3-Kinase Mediated Signaling

The phosphorylated derivatives of phosphatidylinositol (PtdIns) are called phosphoinositides and are regarded as important signaling molecules. Their localization pattern at the membrane marks specific subdomains to which they can selectively recruit cytosolic proteins that harbour one of several known phosphoinositide interaction motifs. These attracted proteins function in the first step of downstream signaling cascades, which finally result in a cellular response.

**Figure 1:**
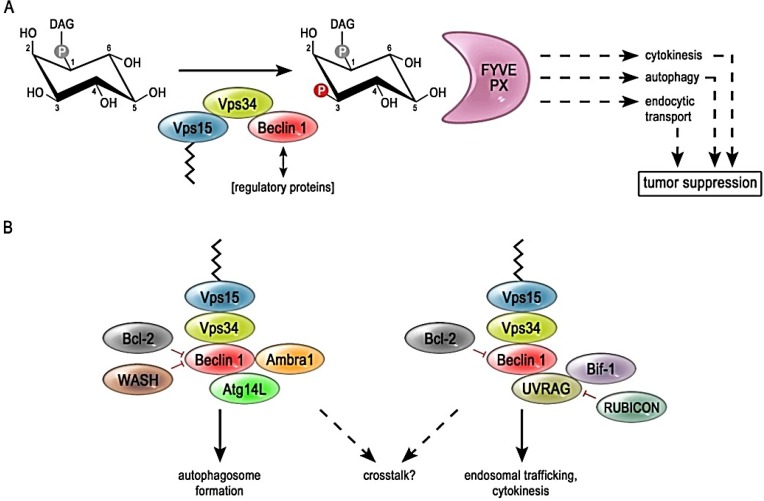
(**A**) Tumor-suppressor function of the PI3K-III complex. The core components of the PI3K-III complex are the lipid kinase VPS34, the membrane-anchoring protein kinase VPS15 and the adaptor protein Beclin 1. Regulatory proteins bind mainly via Beclin 1 to the complex. The substrate of PI3K-III is phosphoinositol, which consists of diacylglycerol-residues (DAG) connected to an inositol ring. VPS34 catalyzes the phosphorylation of the 3'-position of the inositol headgroup, resulting the formation of phosphatidylinositol-phosphate (PtdIns3P). Downstream signaling proteins harbor PtdIns3P-binding domains, like the FYVE-domain or PX-domain. This mode of signaling is an integrative part of the three membrane involution processes that contribute to tumor-suppression: autophagy, cytokinesis and endocytosis. (**B**) Composition and cellular functions of the PI3K-III complexes. The core components of the PI3K-III complex, VPS34, VPS15 and Beclin 1 are essential for all of the PtdIns3P producing sub-complexes. UVRAG and ATG14L bind to Beclin 1 in a mutually exclusive manner and thereby define the two functionally distinct PI3K-III complexes (ATG14L complex and UVRAG complex), which are supposed to be involved in two distinct steps of the autophagic process: autophagosome formation and maturation, respectively. Recent work indicates a possible crosstalk between these complexes. Ambra 1 is part of the ATG14L-containing complex and functions in the early stages of autophagy. The UVRAG-containing complex is stimulated by the interaction with BIF-1, which promotes autophagosome maturation, endocytosis and cytokinesis. Rubicon inhibits the maturation of autophagosomes via its interaction with UVRAG. WASH and Bcl-2 can bind to Beclin 1 and inhibit its function.

This review focuses on phosphatidylinositol 3-phosphate (PtdIns3P), which is mainly generated by the lipid kinase VPS34 (vacuolar sorting protein 34) through the phosphorylation of PtdIns at the 3-hydoxyl group of the inositol ring (Figure 1A). In many cell types, the signaling function of VPS34 and PtdIns3P is reciprocally regulated by other differently phosphorylated derivatives of PtdIns as well as distinct types of lipid kinases.

Three classes of phosphatidylinositol 3-kinases (PI3Ks) have been identified in mammals. They share the capability to phosphorylate the 3-hydoxyl group of the inositol ring, but differ in their substrate specificity and therefore generate distinct phosphoinositide-species [1,2]. These three classes are called PI3K class I, II and III, with PI3K-III being in the focus of this review. However, all three have in common that they are capable to initiate lipid signaling cascades in order to trigger cellular responses [2,3]. The class I PI3K (PI3K-I) is a heterodimeric kinase. It consists of the catalytic subunit p110 and the accessory subunits p85 or p55 [1]. All components exist in different isoforms, which appear to be expressed in different cell types and therefore may have slightly diverging functions. PI3K-I is activated downstream of several receptor tyrosine kinases (RTKs) and G-protein coupled receptors (GPCR), whereupon it preferably generates phosphatidylinositol 3,4,5-trisphosphate (PtdIns(3,4,5)P_3_ or PIP_3_) from phosphatidylinositol 4,5-bisphosphate (PtdIns(4,5)P_2_ or PIP_2_) at the inner leaflet of the plasma membrane [4]. The initial downstream signaling events are often mediated by proteins binding to PtdIns(3,4,5)P_3_ via a Pleckstrin homology (PH) domain [5]. PI3K-I activity initiates cellular survival and proliferative pathways governed by the PH domain-containing kinase AKT and the Ser/Thr-kinase mTOR (mechanistic target of rapamycin), which finally leads to a suppression of autophagy [6,7,8]. The upstream and downstream events in the PI3K-I centered signaling pathway are in many cases associated with tumorigenesis because it is often found to be upregulated caused by hyperactivated RTKs, loss-of-function mutations in the gene encoding PTEN (phosphatase and tensin homolog) or presence of oncogenic forms of the small GTPase RAS [9,10,11].

The PI3K-II is a monomeric kinase, which exists in three isoforms in human cells [12]. In comparison to PI3K-I, its function is just poorly understood [3]. Recent data show, that PI3K-II is activated by RTKs and GPCRs and can generate phosphatidylinositol 3,4-bisphosphate (PtdIns(3,4)P_2_) from PtdIns4P in the context of endocytic trafficking [13]. PI3K-II might also contribute to the PtdIns3P pool required for autophagy [14], even though it is not entirely clear if this depends on a direct production of PtdIns3P from phosphatidylinositol or, alternatively, if it might be generated from PtdIns(3,4)P_2_ by PI 4-phosphatases [1].

PI3K-III is a multi-subunit kinase complex and represents the most conserved PI3K. Furthermore, it is the sole PI3K in yeast and plants [15,16]. The catalytic subunit is VPS34, which exclusively utilizes PtdIns as substrate in order to generate PtdIns3P [15,17,18] (Figure 1).

The produced PtdIns3P has been shown to localize to endosomes and specialized domains of multivesicular bodies [19], phagosomes [20,21], the midbody [22], peroxisomes [23] and pre-autophagosomal structures that are dynamically connected to the endoplasmic reticulum, like omegasomes [24,25]. The generated PtdIns3P recruits PtdIns3P effector proteins, most of which interact via a FYVE (Fab1p, YOTB, Vac1p and EEA1) domain or a PX (Phox homology) domain [5].

The subsequent signaling cascades are involved in downstream events such as the down regulation of growth factor receptors, endocytic signaling, cytokinesis as well as autophagy [26,27,28,29]. These cellular processes play an important role in the prevention of tumorigenesis (Figure 1A). Thus, the PI3K-III mediated signaling might perform its tumor suppressor function by terminating growth factor receptor signaling, as well as by the engulfment of damaged and old organelles in autophagosomes, or through prevention of bi-nucleation and genome instability by ensuring a correct cytokinesis [30]. However, in this context it is important to point out, that autophagy in general functions as a cytoprotective mechanism under stress conditions. But this also means that autophagy can promote tumor growth and resistance to chemotherapy in already established tumor cells and is therefore often attributed as “double-edged sword” [31,32,33].

In order to accomplish full enzymatic and biological activity, VPS34 associates with further regulatory proteins. The core components are the putative protein kinase VPS15 (p150) and the multivalent adaptor protein Beclin 1 (Atg6/Vps30) [26,34]. The myristoylated VPS15 binds via its protein kinase domain to VPS34 and functions as membrane-binding factor for the lipid kinase [15,35]. Furthermore, the WD domain of VPS15 forms a seven-bladed propeller resembling that of typical G-beta subunits [36], which is in line with the finding that the G-alpha protein Gpa1 binds to VPS15 in the context of VPS34-dependent pheromone signaling at yeast endosomes [37].

Beclin 1 interacts with a growing list of transiently associated accessory factors, giving rise to different subunit compositions that specify the localization, activity and physiological context of VPS34-catalyzed PtdIns3P production [26].

## 2. Modulation of PI3K-III Complex Activity through Different Subunit Compositions

VPS34, VPS15 and Beclin 1 are regarded as the core components of the PI3K-III complex and are essential for the catalytic activity as well as physiologic function of the complex within the cell. However, a growing list of accessory proteins of PI3K-III that function as positive or negative regulators of PtdIns3P-production have been described to play an important role as well (Figure 1B). Most of these regulative proteins contact the PI3K-III complex via Beclin 1 [26,34,38]. Therefore, Beclin 1 itself is tightly regulated by competing interaction partners, different subcellular localizations [38], phosphorylation [39] as well as ubiquitination (see Section 3.1, Section 3.2, Section 3.3, Section 3.4 and Section 3.5).

In yeast, two major complexes have been identified in addition to the already mentioned Gpa1-containing assembly in pheromone signaling. The two complexes contain Vps34, Vps15 and the Beclin 1-homolg Vps30(Atg6). While complex I additionally contains Atg14 and Atg38, complex II exclusively harbors Vps38 instead [40,41]. While complex I is required for macroautophagy and selective autophagy [23,40,41,42], the Vps38-containing complex II is involved in vesicular protein sorting [41,42].

A similar mechanism is conserved in mammalian cells, where Atg14L(Barkor) and the Vps38-homolog UVRAG (ultraviolet irradiation resistance-associated gene) are mutual exclusive constituents of two distinct PI3K-III complexes [43,44,45,46]. The Atg14L-containing complex has a central function in autophagy [43,45,46]. Atg14L targets the PI3K-III complex to the endoplasmic reticulum (ER) [47], where it binds to Syntaxin 17 at the ER-mitochondria contact sites [48], that are one possible source for autophagosomal membranes. Atg14L can sense and maintain membrane curvature of PtdIns3P-enriched membrane regions [49] and therefore may function to establish and stabilize the PI3K-III complex at the nascent autophagosome. Furthermore, the interaction of Atg14L with Beclin 1 controls the accessibility of certain residues within Beclin 1 required for the stimulation of autophagy by phosphorylation [50].

Another component of the autophagy-related complex is Ambra 1 (activating molecule in Beclin 1-regulated autophagy) [51]. Ambra 1, for which no yeast homolog has been identified so far, is a WD40-protein that interacts with Beclin 1 [51]. It supports PtdIns3P production by stabilizing the interaction of VPS34 with Beclin 1. Ambra 1 influences autophagy also by other mechanisms. Ambra 1 binds to the dynein light chain 1 (DLC1) at the dynein motor complex under normal conditions, where it also concentrates Beclin1 [52]. Upon induction of autophagy, ULK1 (Atg1) phosphorylates Ambra 1, which is released from the motor complex then and transported to the ER, where it contributes to the process of autophagosome formation [52]. Moreover, Ambra 1 has been shown to be capable of binding LC3 at least in the context of mitophagy [53]. Ambra 1 is also involved in several ubiquitination-reactions that regulate different aspects of autophagy (see Section 4.1, Section 4.2 and Section 4.3).

It is important to note, that the components of the PI3K-III complex are also involved in different pathways as well. Ambra 1 is linked to apoptosis and cell cycle control [54]. Atg14L interacts with SNARE-associated protein Snapin and contributes to the coordination of endosome maturation and endocytic trafficking [55].

In general, the partial overlap of mammalian factors involved both in endosome biogenesis and maturation as well as the formation of autophagosomes has become more evident in recent years [56,57,58]. This is especially evident in the UVRAG-containing PI3K-III complex. In contrast to yeast Vps38, UVRAG is involved in membrane trafficking events contributing both to phagophore maturation during autophagy as well as control of endosome formation, size and maturation [45,46]. Beclin 1—bound UVRAG can interact with the class C Vps complex at endosomes [59]. This interaction stimulates the GTPase activity of Rab7 and thereby drives the fusion of autophagosomes with late endosome/lysosomes, which results in an accelerated delivery and degradation of autophagic cargo. As a separate function, the class C Vps—bound UVRAG complex enhances also endosome-endosome fusion events, resulting in rapid degradation of endocytic cargo [59]. Furthermore, it regulates the trafficking of the essential autophagy factor Atg9 [60].

The UVRAG-associated PI3K-III complex components display also a dual function. Endophilin B1/Bif-1 (Bax-interacting factor 1) supports autophagy by eliciting local Vps34 activity and PtdIns3P-production [61] but also as possible crescent driving force during autophagy due to its membrane sculpturing abilities [62,63] as well as its role in trafficking of Atg9 [60].

In contrast, the UVRAG-associated RUB-domain protein Rubicon is regarded as a negative regulator of autophagy, which inhibits VPS34-activity [45,46,64]. Bif-1 and Rubicon also influence endocytic trafficking. Bif-1 promotes endocytic degradation of NGF (nerve growth factor) [65] and EGF (epidermal growth factor) [66], while Rubicon is linked to endosome maturation via its interaction to the endosomal GTPase Rab7 [67].

Another inhibitory protein that blocks PI3K-III function in general is the proto-oncogene and anti-apoptotic Bcl-2 (B-cell lymphoma 2) [68,69,70]. Bcl-2 is a Beclin 1 binding protein that interacts via its linker region between the BH3 and BH4 domains with the B3 domain of Beclin 1 [71]. It has been suggested that Bcl-2 could prevent the oligomerization of Beclin 1 and therefore would hamper the assembly of the active VPS34 complex, which results in a block of autophagy [72,73,74,75]. The interaction of Beclin 1 and Bcl-2 is regulated by several mechanisms, which involve titration by competing binding partners, inhibitory phosphorylation of Beclin 1 and Bcl-2 [39], as well as ubiquitination (see Section 3.3 and Section 3.4).

The functional interplay of these distinct PI3K-III subcomplex compositions as well as the regulation of key components by ubiquitination and SUMOylation will be discussed in detail in following sections of this review. We will highlight the direct involvement of ubiquitination or SUMOylation events in the function of each example, but also point out observations that might link the corresponding modifying enzyme to other parts of autophagy regulation in order to indicate possible different layers of complexity.

## 3. Regulation of Beclin 1 via Different E3 Enzymes and Distinct Ubiquitin Modifications

Beclin 1 functions as the central adapter module of VPS34 within the PI3K-III complex, because it interacts with most additional binding partners [38,76]. Similar to other central proteins of signaling or transport pathways, like e.g., p53 [77,78], PTEN [79,80], the androgen receptor [81] and peroxisomal targeting signal (PTS)-receptors [82,83], Beclin 1 is modified by different ubiquitin signals. Accumulating evidence in recent years indicates that Beclin 1 is the substrate of versatile distinct ubiquitination modifications.

### 3.1. Inhibition of the Deubiquitinating Enzymes USP10 and USP13 Causes Ubiquitination of Beclin 1

**Figure 2:**
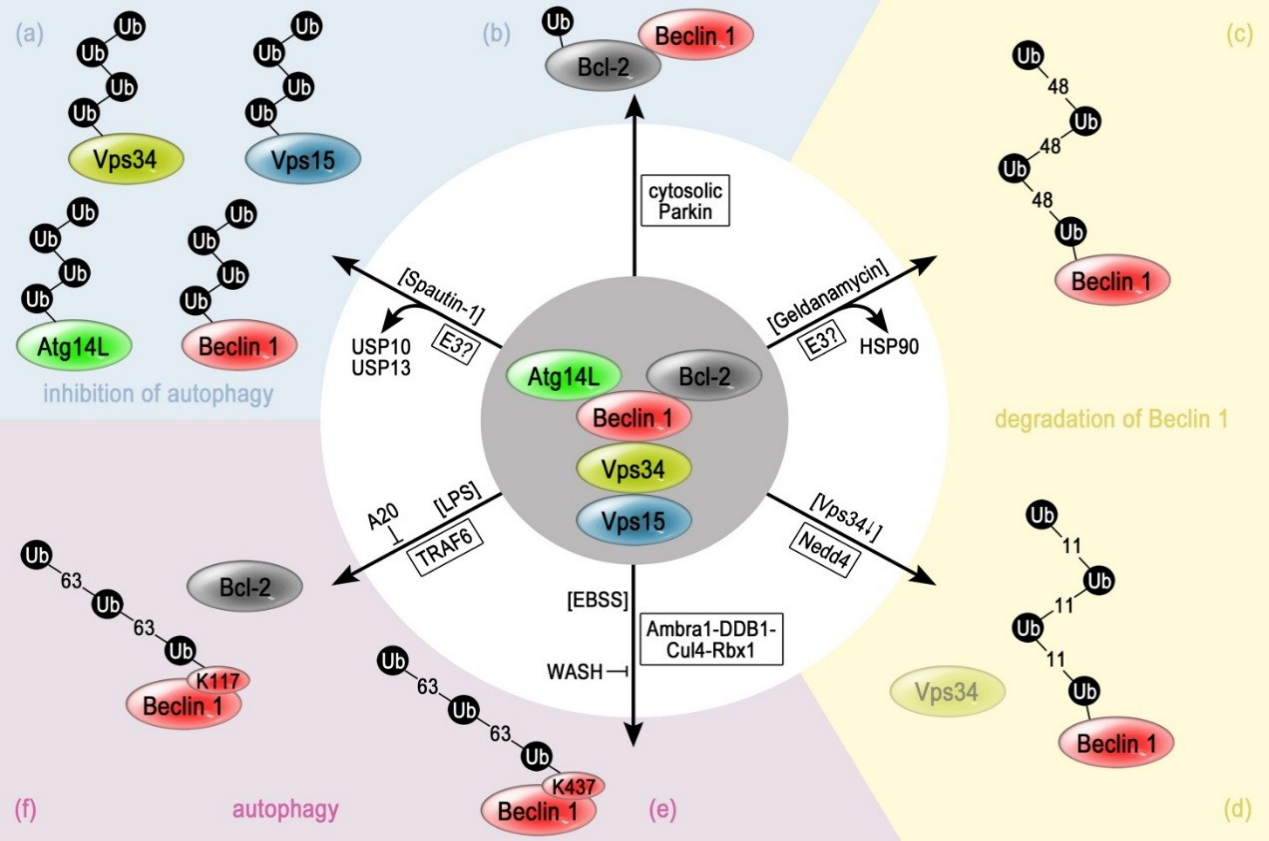
Regulation of Beclin 1 by ubiquitination. The PI3K-III core complex constituent Beclin 1 is substrate for several different ubiquitination machineries. (**a**) Treatment of mouse embryonic fibroblasts (MEFs) with the autophagy-inhibitor Spautin-1 inactivates the Beclin 1—associated deubiquitinating enzymes USP10 and USP13. This leads to polyubiquitination by unkown E3 enzymes and subsequent proteasomal degradation of Beclin 1, VPS34, VPS15 and ATG14L, which finally results in a block of autophagy; (**b**) Another possibility to abrogate autophagy is the monoubiquitination of the Beclin 1-interacting protein Bcl-2. In HeLa cells, the inhibitory factor Bcl-2 can be modified by the E3 RBR-type ligase Parkin in the cytosol. This results in a higher stability of Bcl-2 and therefore in a more stable interaction with and inhibition of Beclin 1; (**c**) Treatment of RAW267.4 cells with geldanamycin abrogates the interaction of Beclin 1 to the chaperone HSP90. The result is the modification of Beclin 1 by unknown E3 enzymes with Lys48-linked polyubiquitin chains and subsequent degradation; (**d**) The cellular level of Beclin 1 can also be reduced in HeLa cells, when VPS34 is downregulated by siRNA treatment. Beclin 1 is polyubiquitinated by the HECT-type Nedd4 with Lys11-linked chains, resulting in its proteasomal degradation; (**e**) Positive regulation of Beclin 1 by Lys63-linked ubiquitin chains has been reported in two cases. MEFs incubated in starvation medium display modified Beclin 1 that has been polyubiquitinated by the cullin-ligase complex consisting of the ligase Rbx1, as well as adaptor proteins CUL4, DDB1 and Ambra 1. This regulatory mechanism is inhibited when Beclin 1 is bound by WASH; (**f**) In macrophages recognizing LPS, Beclin 1 is ubiquitinated by the RING-type ligase TRAF6. The modification of Lys117 abrogates the association with Bcl-2 and thereby promotes autophagy. This mechanism is counteracted by the deubiquitinating enzyme A20, which removes the Lys63-linked polyubiquitin chain of Beclin 1.

A systematic screen for chemical compounds that are able to block autophagy identified Spautin-1 (specific and potent autophagy inhibitor-1) [84]. Spautin-1 treatment of mouse embryo fibroblasts (MEFs) caused ubiquitination and destabilization of Beclin 1 as well as of ATG14L, VPS15 and VPS34 [84]. As a consequence, the overall PtdIns3P production was reduced and autophagy blocked [84]. The linkage of the ubiquitin chain and the responsible E3 enzyme are not known yet. Molecular analysis revealed that Spautin-1 directly inhibits the activity of the deubiquitinating enzymes (DUBs) ubiquitin specific protease 10 (USP10) and USP13. Moreover, USP13 directly interacts with Beclin 1 in untreated cells, while it dissociates from Beclin 1 after addition of Spautin-1 (Figure 2a). Therefore, these findings indicate, that USP13 and USP10 protect Beclin 1 from being ubiquitinated and degraded [84]. Currently, it is not clear if they also directly protect the other PI3K-III complex components or if their degradation upon Spautin-1 treatment is caused indirectly by a destabilization due to the breakdown of Beclin 1.

It has been shown in yeast [41] as well as mammalian cells [85] that reduction of Vps30/Beclin 1 levels causes instability of other PI3K-III complex subunits. Another interesting observation made in the Spautin-1 study is that knock-down of VPS34 and Beclin 1 causes instability of USP10 and USP13, which indicates the existence of a regulatory feedback loop [84]. Moreover, the finding that Spautin-1 leads to the degradation of the central PI3K-III components in the context of autophagy, suggests that possibly this mechanism might also be relevant in the context of endocytosis and cytokinesis, which, however, needs further investigation. Further studies have demonstrated, that Spautin-1 shows enhancing effects in the treatment of chronic myeloid leukemia and ovarian cancer cells, when combined with other drugs [86,87].

Another possible connection of USP10 to autophagy derives from the finding that USP10 can be co-immunoprecipitated with ULK1 [88]. ULK1 is a protein kinase involved in the induction of autophagy. ULK1 phosphorylates not only the mTOR-inactivator AMPK (AMP-activated protein kinase) [89], but also Beclin 1 [90,91] and Ambra 1 [52,92] in order to induce downstream autophagic signaling. However, a direct link of USP10-mediated protection of ULK1 and phosphorylation of Beclin 1 and Ambra 1 has not been demonstrated yet. Moreover, it has been shown that ULK1 assembles with VPS34 onto EXO84, an exocyst complex subunit, after the starvation-induced activation by the small GTPase RALB in an USP33-depenent manner [93,94].

It is important to note, that USP10 has been identified as a deubiquitinating enzyme for p53 [95], especially as the interaction of p53 and Beclin 1 has been suggested to regulate the cellular decision on the induction of apoptosis or, alternatively, autophagy in embryonal carcinoma cells [96].

These potential functional links between the RALB-EXO84-ULK1-assembly, as well as USP10, p53 and VPS34-Beclin 1—complexes strongly suggests the existence of a regulatory network that governs the dynamic interplay of these different signaling hubs.

### 3.2. Lys48-Linked Polyubiquitination of Beclin 1 upon Inhibition of HSP90

HSP90 (heat shock protein 90) is a molecular chaperone, which assists in protein stabilization by aiding protein folding [97]. Furthermore, HSP90 has been reported to be involved in controlling Beclin 1 stability and thus VPS34 activity in monocytes [98]. It has been shown that HSP90 can be inhibited by the benzoquinone ansamycin antibiotic geldanamycin [99]. Interestingly enough, Geldanamycin prevents the interaction between HSP90 and Beclin 1, which results in the formation of Lys48-linked ubiquitin chains on Beclin 1 (Figure 2c) [98]. The Lys48-linked polyubiquitination primes Beclin 1 for degradation by the 26S proteasome and thereby reduces the Beclin 1 protein level [98]. It will be interesting to elucidate if the stability of the other PI3K-III complex members is also affected, as in the case of Spautin-1—mediated inhibition of USP10 and USP13 [84] and if the control of Beclin 1 stability is mediated by HSP90 in general or whether this represents a regulatory event specifically developed in phagocytic cells.

While the E3 ligase that targets Beclin 1 in absence of the interaction to HSP90 is not known, several other HSP90 clients have been reported to be substrates of the E3 ligase Cul5 [100,101]. However, if Cul5 also targets Beclin 1 for degradation is not known. Moreover, the interaction of Beclin 1 with p53 in embryonic carcinoma cells has been found to induce Lys48-linked polyubiquitination and proteasomal degradation of Beclin 1 [96]. However, whether this similar kind of modification can be regarded as an indication that p53 and HSP90 are both involved in the same regulatory context with opposing roles concerning Beclin 1 stability is not clear. Still, this potential link could be worth testing, as p53 has recently been shown to counteract the ATPase activity of HSP90 [102,103].

Moreover, HSP90 is a well-known molecular target for anti-cancer agents. Because HSP90 can also stabilize several transcription factors, cell-cycle regulatory proteins or RTKs that are potentially mutated in tumor cells, it is inhibited by chemical compounds as part of anti-cancer therapies [104,105]. Therefore, the functional contribution of HSP90 has to be seen in a context-dependent way. While it stabilizes the tumor-suppressor Beclin 1 in healthy cells, it also protects potential oncogenic proteins from being degraded.

### 3.3. Lys11-Linked Polyubiquitination of Beclin 1 by Nedd4

The HECT (homologous to the E6-AP carboxyl terminus)-type ligase Nedd4 (neural precursor cell-expressed developmentally down-regulated protein 4) can polyubiquitinate Beclin 1 with Lys11-linked chains (Figure 2d) [106]. Nedd4 can influence the stability of Beclin 1 in two ways in HeLa cells [106]. The interaction between Beclin 1 and Nedd4 is required for the ubiquitination and proteasomal degradation of Beclin 1 and therefore controls its steady-state level. Moreover, when the expression of VPS34 is down-regulated by siRNA, the polyubiquitination and degradation of Beclin 1 by Nedd4 is strongly enhanced [106]. Therefore, Nedd4 could be regarded as part of a quality control mechanism that targets free Beclin 1. In this context, it is interesting to note that the deletion of Vps34 in yeast cells [41] or knock down of VPS34 in HeLa cells [85] reduces the cellular level of Beclin 1. However, it is not clear if these processes also involve Lys11-linked polyubiquitination or require Nedd4-related enzymes.

In a general context, it is important to note that Nedd4 has also been found to be part of the LC3-interaction map of basal autophagy [107]. A systematic RNAi analysis showed that the depletion of Nedd4 results in an increase of the steady-state level of the membrane-bound LC3-II upon inhibition of the vacuolar type H(+)-ATPase by bafilomycin A1 treatment. This strongly indicates that Nedd4 functions in the down-regulation of basal autophagy [107].

Moreover, Nedd4 has been described to ubiquitinate the lipid phosphatase PTEN, which is responsible for the conversion of PtdIns(3,4,5)P into PtdIns(4,5)P [108]. PTEN terminates the proliferative and growth mediating pathways controlled by PI3K-I, AKT and mTOR in order to relieve the AKT/mTOR-mediated suppression of autophagy [1,3,109,110]. One direct target of AKT is Beclin 1, which is inhibited by this phosphorylation event, resulting in a block of autophagy [111]. Therefore, PTEN positively regulates autophagy in general by quenching the AKT signal [112]. Nedd4 has been assigned to counteract this effect via two mechanisms: firstly, by polyubiquitinating PTEN and thereby tagging it for proteasomal degradation [113] and secondly, by supporting the AKT co-activator TRAF3 (tumor necrosis factor receptor—associated factor 3) via non-proteolytic Lys63-linked ubiquitin chains [114].

Interestingly, Nedd4 can function as a negative regulator of p53 [115]. It stabilizes the ubiquitin-ligase Mdm2 by attaching Lys63-linked chains to it, which results in a higher degradation rate of p53 via Mdm2-catalyzed ubiquitination [115]. This could be relevant for the interplay of Beclin 1 and p53, as both are central decision factors for the balance between autophagy and apoptosis induction [116,117]. The stability of both Beclin 1 and p53 is supposed to be regulated reciprocally, in a direct manner by involving a direct interaction between Beclin 1 and p53 [96] or indirectly via the Beclin 1—dependent deubiquitinating enzymes USP10 and USP13 [84,95] (see Section 3.1 and Section 3.2). Therefore, Nedd4could be a potential therapeutic target for the treatment of human cancers [118].

Taken together, these findings indicate that oncogene Nedd4 might function as a negative regulator of the Beclin 1—p53 axis and thereby inhibits the basal protective function of both apoptosis and autophagy.

### 3.4. Lys63-Linked Polyubiquitination of Beclin 1 by TRAF6

Beclin 1 is a substrate of the RING-type ligase TRAF6 (tumor necrosis factor receptor—associated factor 6) in macrophages [119]. These cells harbor Toll-like receptors (TLR) at their plasma membrane, which play an important role in innate as well as adaptive immunity by recognizing conserved molecular patterns of pathogens. The binding of bacterial lipopolysaccharides (LPS) to TLR4 triggers a signaling cascade, which induces phagocytosis of the pathogen as well as autophagy pathways as a key response to multiple stressors [120,121]. It has been demonstrated that the intracellular TLR4-adaptor proteins Trif and MyD88 function as a direct link to PI3K-III signaling via their interaction to Beclin 1 [122]. Upon activation of TLR4 by LPS, they disrupt the interaction between Beclin 1 and its inhibitor Bcl-2 [122]. In addition, another possibility to promote the dissociation of Beclin 1 from Bcl-2 is induced by recruiting the E3 enzyme TRAF6 to Beclin 1 [119]. TRAF6 catalyzes the formation of a Lys63-linked polyubiquitin chain at Lys117 within the BH3 domain of Beclin 1 (Figure 2f) [119]. This chain prevents Bcl-2 from binding to the BH3 domain and therefore enables the Beclin 1-dependent induction of autophagy [119,123].

The TRAF6-cataylzed ubiquitination of Beclin 1 is opposed by the deubiquitinating enzyme A20 [119]. A20 hydrolyzes the peptide-bond between Beclin 1 and ubiquitin, which enables again binding of Bcl-2 and therefore antagonizes the induction of autophagy. This is especially evident when A20 is overexpressed.

It is interesting to note that the described mechanism resembles the principle by which the Beclin 1—Bcl-2 interaction is also disrupted via phosphorylation of the corresponding interaction interfaces [39]. Dissociation of Bcl-2 from Beclin 1 can also be found after a direct phosphorylation of the Beclin 1 BH3 domain (Thr119) by the kinase DAPK (death associated protein kinase) [124], which results in increased autophagy. Future work might elucidate, if the phosphorylation of Thr119 could have a priming function for Beclin 1 ubiquitination within the BH3 domain, because certain E3 ligases, like TRAF6 [125], are known to have phosphorylation requirements as priming factor. Therefore, it will be interesting to investigate if a possible crosstalk between phosphorylation and ubiquitination of Beclin 1 exists.

The LPS/TLR4-induced ubiquitination of Beclin 1 in macrophages is not the only connection of TRAF6 to autophagic pathways. TRAF6 participitates in early stages of autophagic signaling by modifying UKL1 with a Lys63-linked polyubiquitin chain [92], which has, as discussed below (see Section 4.1), a stimulatory effect on autophagy induction by ULK1. Furthermore, work from *Drosophila melanogaster* and mammalian cells has uncovered a link between oxidative stress and autophagy induction [126]. Autophagy is initiated by ROS (reactive oxygen species)-stimulated JNK1-signaling. Recent work indicates that TRAF6 associates with the autophagic scaffold protein Atg9, which is an important regulatory target of ULK1 [127], in order to activate JNK1 by an unknown mechanism [126].

TRAF6 also catalyzes the Lys63-linked polyubiquitination of the kinase TAK1 (transforming growth factor β—activated kinase 1, which can phosphorylate and activate JNK1 [128,129]. JNK1 is known to modify VPS34 activity through phosphorylation of Bcl-2 [130].

In summary, TRAF6 might activate VPS34 by interfering with the amount of Bcl-2 that can bind to Beclin 1, either directly by ubiquitinating the Bcl-2 binding motif in Beclin 1, or indirectly by supporting the JNK1-dependent phosphorylation of the corresponding domain in Bcl-2 that is required for the interaction with Beclin 1 [39,119,130].

A different contribution of TRAF6 to autophagic processes concerns its involvement in the degradation of the midbody ring [131]. Here, TRAF6 ubiquitinates the midbody-localized protein KIF23/MKLP1. Furthermore, TRAF6 associates with the ubiquitin-binding autophagy receptor SQSTM1/p62 and the interacting adaptor protein WDFY3/ALFY, resulting in the degradation of the midbody ring structure by autophagy [131]. These studies show that TRAF6 is involved in different aspects and sometimes even in several functionally interconnected circles required in early steps of autophagy.

### 3.5. Parkin Catalyzes the Monoubiquitination of the Beclin 1 Inhibitor Bcl-2

The RBR (RING-between-RING)-type ubiquitin ligase Parkin, which is frequently found to be mutated in the neurodegenerative Parkinson’s disease (PA) [132,133], has an important role in the regulation of the association of Beclin 1 with Bcl-2. In contrast to the effect triggered by TRAF6, DAPK and JNK1 (see Section 3.3), the Beclin 1—Bcl-2 interaction is strengthened by Parkin [134]. Parkin monoubiquitinates Bcl-2 (Figure 2b), which results in a higher stability of the Beclin 1-inhibitor [134]. Currently, it is not clear how monoubiquitination of Bcl-2 contributes to its stability. In general, monoubiquitination (monoUb) is capable to induce conformational changes within the target protein that might result in an altered subcellular localization or might block the interaction to potential destabilizing factors such as degradation-linked E3 enzymes [135]. In the end, the stabilized monoUb-species of Bcl-2 seems to be more effective in the binding of Beclin 1 and inhibition of autophagy, possibly due to a prolonged association with the PI3K-III complex [134].

Currently, it is not known if the Parkin-mediated monoubiquitination and stabilization of Bcl-2 is restricted to the context of autophagy or if it also affects endocytic sorting or cytokinesis.

While cytosolic Parkin seems to have a negative regulatory role concerning (macro)autophagy [134], it has been reported that it has a stimulatory role in the selective autophagic degradation of mitochondria [136], where it cooperates with the PI3K-III component Ambra 1 (see Section 4.2).

In the context of the occurrence of Parkinson’s disease, it has been reported that Beclin 1 can also interact directly with Parkin [137]. This interaction promotes the degradation of alpha-synuclein [137], a target protein of Parkin [138]. However, the molecular mechanism is not resolved. In PC12 (pheochromocytoma of rat adrenal medulla) cells Beclin 1 has been found to target Parkin to mitochondria in order to induce mitophagy [139]. The question, if Beclin 1 is ubiquitinated by Parkin remains to be answered. Furthermore, Beclin 1 has been found to interact with the Parkin-binding and Parkinson-associated PINK1 (PTEN-induced putative kinase 1) [140].

Another interesting connection between Beclin 1 and Parkin might link Beclin 1 to Alzheimer’s disease (AD). In animal AD models, Parkin ubiquitinates Amyloid-beta and stimulates the Beclin 1-dependent autophagicclearanceof ubiquitinated Amyloid-beta as well as of defective mitochondria [141,142,143]. This Beclin 1—dependent clearance of intraneuronal Amyloid-beta may counteract extracellular plaque deposition and restore neurotransmitter balance [144,145,146]. In line with this, the co-localization and interaction of Beclin 1 and Parkin have been described to facilitate the clearance of Amyloid-beta and improve cognitive performance [147]. This interplay is enhanced by tyrosine kinase inhibitors [147]. Conversely, accumulation of Amyloid-beta has an oligomerization-dependent effect on the functionality of Beclin 1 [148]. Monomeric Amyloid-beta triggers the JNK1-mediated phosphorylation of Bcl-2 and therefore frees Beclin1 in order to induce autophagy, while Amyloid-beta oligomers block this phosphorylation, which strengthens the Bcl2-Beclin 1 interaction and results in autophagy-suppression and apoptosis [148]. Similar to the situation with the Beclin 1-Parkin interaction in the context of PA, it is not known if ubiquitination of Beclin 1 itself plays a role in AD.

### 3.6. Lys63-Linked Polyubiquitination of Beclin 1 by the Ambra 1-Containing Cullin-RING-Ligase

Beclin 1 has been found to be a substrate of a multi-subunit ligase of the Cullin-RING (CR)-family. These complex ligases contain the RING-type E3 ligase Rbx1 as catalytic subunit, but also the scaffold protein Cul4 as well as different substrate-selective adaptors [149]. Most interestingly, Ambra 1 had been shown to be a Cul4-associated protein, which is indicated by an alias of Ambra 1, DCAF3 (DDB1- and Cul4-associated factor 3) [150,151]. DCAF substrate receptors confer the substrate specificity of complex E3 ligases [151,152].

More recently, the Ambra 1-containing Rbx1/Cul4-ligase complex has been demonstrated to ubiquitinate Lys437 of Beclin 1, which finally results in the formation of a Lys63-linked polyubiquitin chain (Figure 2e) [153]. This ubiquitination is initiated when MEFs (mouse embryonic fibroblasts) are shifted to starvation medium and has a positive impact on starvation-induced autophagy. This ubiquitin-modification might function as a scaffold that triggers the assembly of the required binding partner. Consequently, the interaction to VPS34 is stronger and VPS34 is more active than in cells that harbor an ubiquitination-resistant variant of Beclin 1 [153]. Therefore, the ubiquitination by the Ambra 1-containing cullin ligase is important for autophagy under these conditions. The interaction between Beclin 1 and Ambra 1 is competitively disrupted by WASH (Wiskott-Aldrich syndrome protein (WASP) and SCAR homologue). WASH is a member of the WASP family [154] and has been known to have an important function in endosome sorting [155]. This newly identified inhibitory function in the context of autophagy is based on the concept that WASH prevents the interaction with the Ambra 1-containing ligase complex [153]. Recently, an additional mode of autophagy inhibition has been suggested, namely the WASH-triggered and RNF2-catalyzed polyubiquitination of Ambra 1 [156] (see Section 4.1).

## 4. Involvement of Ambra 1 in Ubiquitination-Dependent Processes

The WD40-protein Ambra 1 has been identified as a Beclin 1 binding partner, which functions as a crucial activator of the initial steps of phagophore formation in the nervous system [51]. Cells bearing loss-of-function mutants of Ambra 1 display impaired autophagy, accumulate ubiquitinated proteins and are characterized by excessive apoptosis [51].

### 4.1. Ubiquitination of Ambra 1 by RNF2 and Its Role as Rbx1/Cul4-Ligase Complex Adaptor

**Figure 3:**
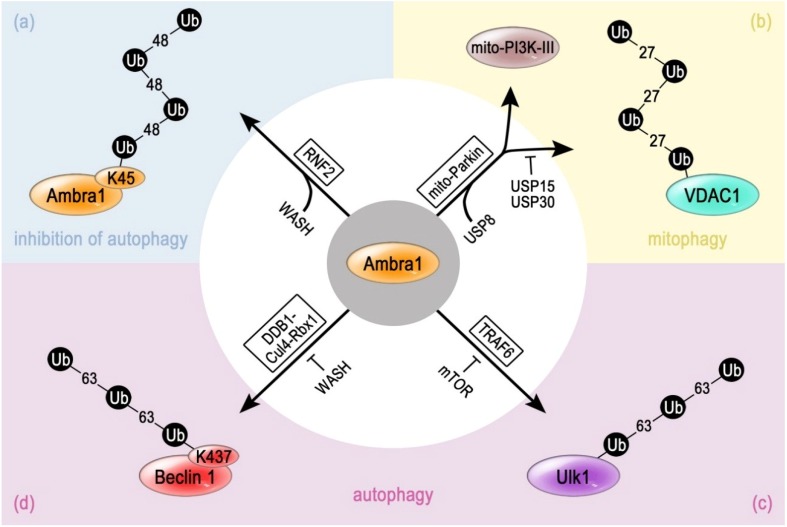
Involvement of Ambra 1 in ubiquitin-dependent processes. (**a**) The position Lys45 of Ambra 1 can be polyubiquitinated with a Lys48-linked chain by the RING-type ligase RNF2, resulting in its degradation. RNF2 is targeted to the PI3K-III complex via its interaction to the autopahgy inhibitor protein WASH; (**b**) Ambra 1 can support the ubiquitination of other target proteins. In HEK293 and mouse brain cells, mitochondrial Ambra 1 promotes mitophagy in two ways. It attracts Beclin 1 and the PI3K-III complex, but possibly also interacts with the RBR-type E3 ligase Parkin. Parkin ubiqitinates mitochondrial proteins like VDAC1 with Lys27-linked polyubiquitin chains, and thereby is supposed to mark the organelle for recognition by autophagy-receptors. In general, this process can be blocked by the deubiquitinating enzymes USP15 and USP30, which deubiquitinate mitochindrial targets of Parkin, or enhanced by USP8, which deubiquitinates Parkin; (**c**) In MEF cells, Ambra 1 can bind the RING-type E3 enzyme TRAF6. This stabilizes the association with ULK1, which can be polyubiquitinated by TRAF6 with Lys63-linked Ub-chains. This process supports autophagy. Phosphorylation by mTOR inhibits this mechanism; (**d**) In MEF cells, Ambra 1 was found to be part of the cullin-ligase complex consisting of the enzymatic subunit Rbx1 as well as the accessory constituents DDB1 and Cul4. Ambra 1 may function as a substrate adaptor because it interacts with Beclin 1, which is then polyubiquitinated via Lys63-linked chains. This modification promotes the progress of autophagy.

Nutrient deprivation stimulates the induction of autophagy and triggers the association of Ambra 1 with the PI3K-III core complex via its interaction to Beclin 1 [51]. One important role of Ambra 1 within the PI3K-III complex of MEFs is its function as Cullin-ligase adaptor protein [153] (see Section 3.5). This complex ligase, consisting of Rbx1, DDB1, Cul4 and Ambra 1, catalyzes the non-proteolytic Lys63-linked polyubiquitinylation of Beclin 1, resulting in an enhanced PtdIns3P production by VPS34. This process is blocked by the interaction of WASH with Beclin 1 [153] (Figure 3d).

Recently, another molecular mechanism underlying WASH-mediated inhibition has been put forward. WASH functions also as a targeting factor for the RING-type E3 ligase RNF2 (ring finger protein 2)/Ring1b) [156]. RNF2 modifies Lys45 of Ambra 1 with a Lys48-linked polyubiquitin chain, which results in the proteasomal degradation of Ambra 1 and the downregulation of autophagy [156] (Figure 3a). Moreover, cells deficient for RNF2 display an enhanced level of autophagy. Therefore, the Ambra 1-dependent ubiquitination of Beclin 1 linked to the RNF2-mediated ubiquitination of Ambra 1 might well represent a timely defined regulatory loop, which controls autophagy induction and its shutdown via an Ambra 1-centered mechanism.

RNF2 has been known before as a member of the Polycomb group complex (PgC) [157]. The PgC is one of the very few known hetero-trimeric RING ligase complexes [158,159]. Together with the RING-proteins Bmi1 and Ring1a, it acts as an E3 ligase on the histone H2A for its monoubiquitination [160,161], suggesting that RNF2(Ring1b) plays a pivotal role in early development. Furthermore, it has been demonstrated to polyubiquitinate Geminin, a DNA replication inhibitor, in order to maintain the activity of hematopoietic stem cells [162]. In line with its polyubiquitination of Ambra 1 and downregulation of the tumor-suppressor activity of the PI3K-III complex [156], RNF2 also polyubiquitinates the tumor suppressor TP53, leading to tumor formation [163]. If also Bmi1 and Ring1a contribute to the ubiquitination of Ambra 1 remains to be demonstrated. The following studies link Ambra 1 to the execution of ubiquitin-dependent processes that target other key regulator proteins in the context of autophagy (Figure 3).

### 4.2. Cooperation of Ambra 1 with TRAF6 in ULK1 Ubiquitination

An interesting indication for the existence of different layers of interdependent ubiquitination and phosphorylation events in the regulation of autophagy at the PI3K-III complex has been reported recently.

Ambra 1 has been found to be modified by two different kinases with contrary outcome. While phosphorylation of Ambra 1 by mTOR inhibits autophagy, phosphorylation of Ambra 1 by ULK1 elicits autophagy [52,92]. Interestingly, the latter phosphorylation event requires ubiquitin-based signaling to occur.

The E3 ligase TRAF6 has been identified as a binding partner of dephosphorylated Ambra 1 in HEK293 cells [92]. However, Ambra 1 does not serve as the target but as bridging factor to the substrate of the E3, namely the kinase ULK1. Therefore, Ambra 1 may have a similar function as substrate adaptor protein as in the case of the Rbx1/Cul4-ligase complex during ubiquitination of Beclin 1 [153] (see Section 3.5 and Section 4.1). The Ambra 1—bound TRAF6 modifies ULK1 with a Lys63-linked polyubiquitin chain (Figure 3c). This results in a stabilization and self-association of ULK1, which is then able to phosphorylate Ambra 1. This might represent a positive regulation loop [92].

However, this mechanism is blocked when Ambra 1 is phosphorylated by mTOR, which renders Ambra 1 unable to bind TRAF6 and therefore prevents ubiquitination of ULK1 and thereby inhibits autophagy.

Moreover, a recent study uncovered a direct relationship between ULK1 and Beclin 1 [91]. Under amino acid starvation conditions and shutdown of mTOR signaling, ULK1 can phosphorylate Beclin 1 at Ser14 [91], resulting in a higher PI3K-III complex activity. Finally, it would be of interest to elucidate, if also the Lys63-linked polyubquitination of Beclin 1 by TRAF6 observed in macrophages [119] requires Ambra 1 as adaptor protein and if this ubiquitination is preceded by the mentioned ULK1-mediated phosphorylation of both Beclin 1 and Ambra 1.

### 4.3. Functional Interplay of Ambra 1 with Parkin in Mitophagy

Mutations within the E3 ligase Parkin are the most common cause of Parkinson’s disease (PD) [164]. Accumulating work in recent years suggests that Parkin surveys mitochondrial quality by promoting autophagic removal of depolarized mitochondria [165,166,167,168]. Parkin forms an ubiquitin E3 ligase complex with the mitochondrial kinase PINK1, and the multifunctional protein DJ-1 in order to promote the degradation of unfolded proteins [169]. Moreover, Parkin modifies the signaling molecule NEMO with linear, non-proteolytic Ub-chains in order to contribute to mitochondrial integrity [170]. Therefore, the concept is discussed, that mitochondrial quality control mediated by Parkin might play a critical role in the protection against parkinsonism [133,171].

Parkin-mediated mitophagy involves the translocation of cytosolic Parkin to depolarized mitochondria, which is dependent on PINK1. Subsequentially, Parkin ubiquitinates outer mitochondrial membrane proteins, such as Mitofusin 1, Mitofusin 2 or VDAC1 [165,166,167,168]. At least in the case of the ubiquitination of VDAC1, the attachement of Lys27-linked polyubiquitin chains has been demonstrated [166] (Figure 3b). The accumulating ubiquitinated mitochondrial proteins attract the autophagy-receptor p62 [166,167], which simultaneously binds to ubiquitin and LC3, a protein present on growing phagophores [172].

Finally, the clustered mitochondria are engulfed by autophagosomes. This process is negatively regulated by USP15 [173] and USP30 [174], which deubiquitinate mitochondrial Parkin-targets, while it is supported by USP8, which deubiquitinates Parkin itself [175].

A possible additional mechanistic aspect is indicated by the interaction of Parkin with Ambra 1 (Figure 3) [136]. Ambra 1 is recruited to mitochondria via its association to Parkin. Ambra 1 accumulates around juxtanuclear clusters of depolarized mitochondria, where it activates the PI3K-III complex and triggers the fomation of the phagophore. Therefore, Parkin might stimulate mitophagy also by targeting of Ambra 1 [136,176]. However, overexpression of Ambra 1 can also stimulate mitophagy in an Parkin-independent manner [53]. It is interesting to note, that the important binding partner of Parkin, PINK1, has also been described to interact with Beclin 1 in order to promote autophagy [140]. Moreover, Beclin 1 has been shown to target Parkin to mitochondria in order to stimulate mitophagy in PC12 cells [139].

While translocation of cytosolic Parkin to mitochondria does not depend on Ambra 1, it is blocked by Bcl-2 [177], the inhibitory binding factor of both Beclin 1 and mitochondrial Ambra 1 [69,178]. In contrast, cytosolic Parkin monoubiquitinates and stabilizes cytosolic Bcl-2, which blocks macroautophagy [134] (see Section 3.4). This indicates that Parkin might stimulate macroautophagy and mitophagy in a localization-dependent manner.

It will be important to unveil the functional interconnection of this interaction network between Parkin, Beclin 1, Bcl-2 and Ambra 1.

## 5. Ubiquitination and SUMOylation of VPS34

While Beclin 1 and Ambra 1 have been demonstrated to be involved in several different ubiquitination-dependent regulatory processes (see Section 3 and Section 4), much less is known in case of the lipid-kinase VPS34 itself. The only study that describes ubiquitination and proteasomal degradation of VPS34 detects these modified forms of the lipid-kinase after the cells have been treated with Spautin-1 [84]. This chemical compound inhibits the deubiquitinating enzymes USP10 and USP13, which results in a destabilization of the PI3K-III complex and ubiquitination not only of VPS34, but also of VPS15, Beclin 1 and ATG14L [84] (see Section 3.1; Figure 2).

Recently, VPS34 has been shown to be modified with the ubiquitin-like SUMO 1 (small ubiquitin-like modifier 1) [179] in MCF7 cell lines. SUMOylation is the posttranslational modification of lysine residues with the ubiquitin-related SUMO [180]. SUMOylation also requires an enzyme cascade, which involves the hetero-dimeric E1 Aos1/Uba2, the E2 enzyme Ubc9 and in several cases one of a few known E3 SUMO-ligases [180]. Moreover, the SUMO-moiety can be removed from the target protein by SUMO-deconjugating enzymes [181].

**Figure 4:**
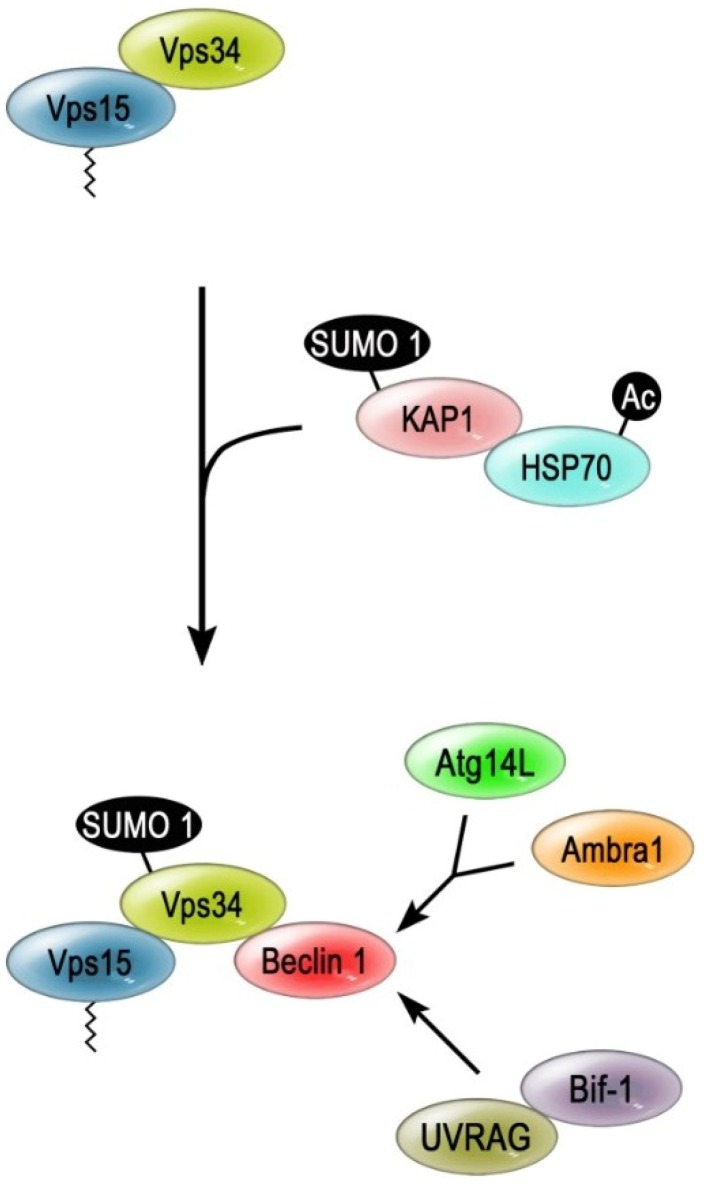
SUMOylation of VPS34. The amino acid starvation of breast cancer (MCF7) cells induces the acetylation (Ac) of HSP70. The acetylated form of HSP70 can bind to the SUMO ligase KAP1 and enables this SUMO-E3 enzyme to modify VPS34. The SUMOylated VPS34 binds more stable to Beclin 1 and the regulatory subunits of the PI3K-III complexes. This fully associated complex promotes the formation of the phagophore and thereby stimulates autophagy.

The SUMO-ligase that targets VPS34 is KAP1 (KRAB-associated protein 1) [179] (Figure 4). KAP1 binds Ubc9 via its RING-like PHD (plant homeo domain) motif, which enables KAP1 to auto-SUMOylate itself in order to be able to stimulate the histone methyltransferase activity of its binding partner SETDB1 [182,183]. The newly identified role of KAP1 in autophagy depends on the stress induced acetylation of the heat shock protein HSP70, because only the HSP70-bound SUMO-ligase associates with its potential target, VPS34, under these conditions [179]. VPS34 is mainly SUMOylated at Lys840, which results in a stronger interaction with the other PI3K-III complex components such as the ATG14L- and UVRAG-containing subcomplexes. Therefore, the SUMOylation of VPS34 has been suggested as a crucial step in the function of the PI3K-III complex of MCF7 cells [179].

In general, cancer cells display an imbalance of the SUMOylation signaling patterns [184]. Ubc9 was found to be overexpressed in ovarian carcinoma specimens, while increased levels of the desumoylating enzyme SENP1 were reported in prostate cancer [184,185,186]. This possible interconnection of SUMO-dependent regulation and cancer has been suggested on the basis of the modification of nuclear proteins. Therefore, finding that the tumor-suppressor activity of the lipid kinase-based signaling of VPS34 is also regulated by SUMOylation adds another potential link of SUMO and the occurrence of cancer. However, the concept that the SUMOylation of VPS34 is indeed directly linked to the prevention of tumorigenesis of healthy cells or the resistance of already transformed cells, seems likely, but requires further investigation.

## 6. Conclusions

Several studies published in recent years clearly demonstrate the important functional role of ubiquitination and SUMOylation events in the dynamic regulation of the PI3K-III complex. Especially the multivalent adaptor protein Beclin 1 has been shown to be ubiquitinated with several different types of ubiquitin chains by different E3 ligases. Moreover, the multi-functional protein Ambra 1 has not only been shown to be ubiquitinated itself, but also to contribute to several other ubiquitin-dependent processes. Further work will be required to elucidate if the detected ubiquitination reactions and E3 enzymes are potentially active within the same cell type or if they represent cell type specific events. Furthermore, the entire published literature so far concentrates on mammalian cells, while it is currently unclear if ubiquitination plays also a similar important role in yeasts and plants.

Based on the already well analyzed phosphorylation reactions that regulate the PI3K-III complex, it is of particular interest to analyze the possible cross-talk between ubiquitination and phosphorylation.

Another interesting aspect concerns the possible different layers of complexity in the regulation of PI3K-III complex function and autophagy in general. As pointed out in each chapter, most of the described E3 enzymes contribute to the regulation of autophagy not only by the ubiquitination of PI3K-III complex components but also in additional ways. It is tempting to speculate that the corresponding E3 enzymes are involved in a cascade-like regulation of different steps in the induction and proceeding of autophagy.

The elucidation of the molecular as well as systemic mechanism of E3 enzymes involved in the regulation of the PI3K-III complex will contribute to the establishment of a dynamic regulatory network, which will be important to understand the complex interplay of its constituents in the occurrence of neurodegenerative diseases and cancer.
